# Seroepidemiology of SARS-CoV-2 Virus in Healthcare Workers before Circulation of the Omicron Sublineages BA.4/BA.5 in Vojvodina, Serbia

**DOI:** 10.3390/vaccines10122168

**Published:** 2022-12-16

**Authors:** Mioljub Ristić, Vladimir Vuković, Aleksandra Patić, Miloš Marković, Vladimir Petrović

**Affiliations:** 1 Department of Epidemiology, Faculty of Medicine, University of Novi Sad, 21000 Novi Sad, Serbia; 2 Institute of Public Health of Vojvodina, 21000 Novi Sad, Serbia; 3 Department of Microbiology with Parasitology and Immunology, Faculty of Medicine, University of Novi Sad, 21000 Novi Sad, Serbia; 4 Department of Immunology, Faculty of Medicine, Institute of Microbiology and Immunology, University of Belgrade, 11000 Belgrade, Serbia

**Keywords:** healthcare workers, seroprevalence, SARS-CoV-2, COVID-19, Vojvodina, Serbia

## Abstract

Healthcare workers (HCWs) are a vulnerable and critical population in the ongoing response to the SARS-CoV-2 pandemic. We aimed to estimate the seroprevalence in HCWs considering all of their previous contacts with the SARS-CoV-2 virus and/or the immunity acquired through their immunization against COVID-19 before the advent of the Omicron variants BA.4/BA.5. Serum samples were collected from 28 March to 10 June 2022. We covered 25% out of all the people who worked in some of the government healthcare centers (primary, secondary, and tertiary level) across the entire Autonomous Province of Vojvodina (Northern Serbia). Two serological tests (Anti-SARS-CoV-2 QuantiVac ELISA and LIAISON^®^ SARS-CoV-2 TrimericS) were used to detect anti-spike IgG antibodies. The overall prevalence of the SARS-CoV-2 antibody among the 6936 HCWs was 92.96% [95% CI 92.33–93.55]. Regarding the type of serological test, there was a statistically significant (*p* = 0.0079) difference of the seropositivity obtained by the LIAISON^®^ SARS-CoV-2 TrimericS (93.87%, 95% CI 92.97–94.69) and Anti-SARS-CoV-2 QuantiVac ELISA (92.23%, 95% CI 91.34–93.06) tests. Seropositivity to SARS-CoV-2 significantly (*p* < 0.0001) increased with the number of SARS-CoV-2 infections combined with the number of doses of the SARS-CoV-2 vaccines received. A vast majority of the HCWs in Vojvodina had detectable levels of antibodies to the spike protein of SARS-CoV-2, but despite this high seropositivity, it is unknown whether this herd immunity among HCWs is protective against the new variants of concern. Further research should evaluate the rates of reinfections and the associated severity of COVID-19 caused by the Omicron sublineages and/or new variants of SARS-CoV-2 among HCWs.

## 1. Introduction

Serological surveillance (serosurveillance) provides estimates of the population-level immunity against (vaccine-preventable) diseases using cross-sectional studies of antibody prevalence [1]. Therefore, a serological survey of specific antibodies against SARS-CoV-2 is helpful to estimate the number of people that have been exposed to SARS-CoV-2 (including asymptomatic and mild COVID-19 cases) and/or have been vaccinated, and to better clarify the dynamics of the epidemic waves [2,3]. Despite the fact that it is still not clear whether the antibodies against SARS-CoV-2 correlate with protective immunity or for how long protective antibody titres will be maintained, serological research of the SARS-CoV-2 antibodies among residents in a certain territory offers the possibility to approximate the number of those who could potentially exhibit immunologic protection against subsequent infections [4]. Seroprevalence studies also provide insights into the real magnitude of the SARS-CoV-2 infections in the community and the extent of the under-detection and under-reporting of COVID-19 cases. Indeed, we have previously shown that in the Northern Serbia, in the Autonomous Province of Vojvodina (with a total population of 1.9 million people), the total number of COVID-19 cases was largely underestimated during the first two waves of the pandemic (the period from March until September 2020) [2]. During this period, only the patients who had signs/symptoms related to COVID-19 were tested using RT-PCR tests (rapid antigen tests were not available at the time). Based on the data of this serological survey, it was estimated that for every RT-PCR confirmed case of COVID-19 there were 39–87 additional infections in Vojvodina. Moreover, in the same study the overall seroprevalence of Vojvodina’s population at the end of September 2020 was estimated to be 16.67% [2]. Finally, in order to estimate the herd immunity to the SARS-CoV-2 virus, seroprevalence studies can obtain the data about the relationships between infection/vaccination, symptomatology, and the subsequent antibody responses for safeguarding the workforce [4]. 

Due to the course of their work and the potential exposed hazards to a SARS-CoV-2 infection, healthcare workers (HCWs) as well as those employed in the healthcare system (staff members) are critical in the ongoing response to the pandemic. Generally, the subjects employed in the healthcare system have a higher risk of a SARS-CoV-2 infection than those from the general population [4,5]. From 6 March 2020, when the first COVID-19 case was confirmed in Serbia until 26 June 2022 (before the confirmed circulation of the Omicron variants BA.4/BA.5), a total of 2,026,045 SARS-CoV-2 infections (primary and reinfections) had been registered in Serbia [6,7], including 449,210 infections in Vojvodina, out of which 19,272 (4.29%) were in subjects employed at healthcare centers across the whole province. However, since many of the infections in HCWs may have been undetected, the true prevalence of the previous SARS-CoV-2 infections in our country among HCWs remains unclear. In addition, due to the expected high risk of COVID-19, especially in those HCWs on the COVID-19 frontline and a significant variation of the reported prevalence worldwide, a seroepidemiological investigation of the antibodies against SARS-CoV-2 is needed across different healthcare settings that includes not only HCWs at the primary healthcare level but also those working in specialized tertiary institutions. Vaccination against COVID-19 in Serbia started on 24 December 2020, and initially covered the elderly population, HCWs, and the residents of long-term care facilities [8,9]. During the previous two and a half years of the pandemic, a majority of the HCWs in Serbia have been vaccinated and many of them have also had COVID-19 (once or several times), with the rates of infections being especially high during the Omicron predominance in 2022, so it is plausible to assume that most of them have developed some level of protection against the SARS-CoV-2.

In order to assess this, we conducted a research aimed at determining the seroprevalence and the associations of different factors with seropositivity to the SARS-CoV-2 virus among medical/non-medical staff at three different healthcare levels (primary, secondary, and tertiary) in Vojvodina, in the spring of 2022, before the circulation of the Omicron variants BA.4/BA.5. 

## 2. Materials and Methods

### 2.1. Study Design and Participants

A cross-sectional study was designed to assess the exposure factors which contributed to the spread of SARS-CoV-2 before the circulation of the Omicron BA.4/BA.5 variants in Vojvodina, Serbia. Serum samples were collected from 28 March to 10 June 2022.

Data obtained in this manner were used to estimate the prevalence of SARS-CoV-2 antibodies in people who worked in some of the government healthcare centers (primary, secondary, and tertiary) covering the entire territory of Vojvodina. Although there was no predefined sample size, a total of 6936 staff members (both medical and non-medical) were included, reflecting 25% out of 27,738 employed adults across healthcare centers in Vojvodina.

All of the participants that were included in the study were asymptomatic. We also included participants who had previous symptomatic COVID-19, but had no symptoms for at least 14 days following the resolution of the disease. In contrast, the study participants were excluded if they reported symptoms of COVID-19 in the 14 days before sampling.

Before enrollment in the study, all of the participants were informed about the aims and purpose of the study and their informed consent was provided. After this, participants were interviewed to collect the following information: their general demographic data, occupation, workplace at the healthcare level, existing comorbidities if any, exposure to the COVID-19 virus at the workplace, the number of previous laboratory-confirmed cases of COVID-19, and their vaccination status (this information was retrieved from the Institute of Public Health of Vojvodina (IPHV) surveillance database), as well as the clinical form (asymptomatic, mild, severe, or critical) of their last episode of COVID-19 (regardless of the number of previous SARS-CoV-2 infections). 

The study participants were invited to be tested for anti-SARS-CoV-2 antibodies. The samples were initially collected at the healthcare centers where the participants worked. Upon sample collection (one day per one site), the samples of all of the participants were promptly transferred to the IPHV, Novi Sad.

### 2.2. Laboratory Testing

The blood samples for the anti-SARS-CoV-2 spike glycoprotein serology were obtained from each participant aseptically using a venepuncture technique. The samples were transported in a cold chain mode to the laboratory of the Centre for Virology at the IPHV, where they were centrifuged and the serum was separated from the clot. Until the moment of serological analysis, the serum samples were adequately stored for one week at +4 to +8 °C, and for longer periods at −20 °C. The serums were tested using a quantitative Anti-SARS-CoV-2 QuantiVac ELISA IgG test (Euroimmun, Lübeck, Germany) (in further text referred as ELISA) or a LIAISON^®^ SARS-CoV-2 TrimericS IgG test (DiaSorin, Saluggia, Italy) (in further text referred as CLIA), both of which measure the total amount of antibodies against the SARS-CoV-2 spike (S) protein.

#### 2.2.1. Anti-SARS-CoV-2 QuantiVac ELISA Test

The ELISA test provides quantitative measurements of the concentration of IgG antibodies against the S1 antigen (including RBD) of SARS-CoV-2 in a broad linear range (using a 6-point calibration curve). The test supports the assessment of the level of immune reaction following a SARS-CoV-2 infection or vaccination with spike-protein-based vaccines. The testing was performed on an EUROIMMUN Analyzer I-2P according to the manufacturer’s instructions [10]. The ELISA test was based on 96-well microplates coated with the recombinant S1 domain of the spike protein of SARS-CoV-2. In the first reaction step, the diluted samples were incubated in the wells. In the case of positive samples, specific IgG antibodies bound to the antigens. To detect the bound antibodies, a second incubation was carried out using enzyme-labeled anti-human IgG antibodies (an enzyme conjugate) that catalyzed a color reaction. In the next incubation, the conjugate reacted with the substrate and a colored product of the reaction was formed. Photometric measurement of the color intensity was performed, which was indicative of the antibodies to SARS-CoV-2 present in the calibrators, samples, or controls. Due to a linear correlation of the results in relative units per milliliter (RU/mL) with the “First WHO International Standard” (NIBSC code: 20/136) [11], the results from the quantitative sample evaluation were converted into standardized units. The resulting concentrations were converted into binding antibody units per milliliter (BAU/mL) by multiplying them by a factor of 3.2. All of the samples with IgG titers below 8.0 RU/mL (<25.6 BAU/mL) were considered negative, values ≥8.0 RU/mL and <11.0 RU/mL (≥25.6 BAU/mL to <35.2 BAU/mL) were considered equivocal, while those with titers equal to or greater than 11.0 RU/mL (≥35.2 BAU/mL) were considered as positive. For the purpose of analysis, the values of titers with <35.2 BAU/mL were considered as seronegative, while the values of ≥35.2 BAU/mL were considered as seropositive to SARS-CoV-2. The sensitivity of this test, in cases when it is used at 10 days and 21 days after symptom onset is 90.3%, and 93.2%, respectively. The specificity of this ELISA test amounted to 99.8% [10].

#### 2.2.2. LIAISON^®^ SARS-CoV-2 TrimericS Test

This test is a new generation of indirect chemiluminescence immunoassay (CLIA) for the detection of IgG antibodies to SARS-CoV-2. 

The testing was performed on a LIAISON^®^ XL Analyzer strictly according to the manufacturer’s instructions [12]. The principal components of the test are magnetic particles (solid phase) coated with a recombinant trimeric SARS-CoV-2 spike protein and a conjugate reagent containing an anti-human IgG mouse monoclonal antibody linked to an isoluminol derivative (isoluminol-antibody conjugate). The trimeric Spike Glycoprotein is the stabilized native form of the SARS-CoV-2 spike protein. It detects a broader repertoire of the neutralizing antibodies, improving the sensitivity and accuracy of the immune status monitoring. During the first incubation, the SARS-CoV-2 IgG antibodies present in the calibrators, samples, or controls bound to the solid phase. During the second incubation, the antibody conjugate reacted with the antibodies to SARS-CoV-2 already bound to the solid phase. Subsequently, the starter reagents were added and a flash chemiluminescence reaction was thus induced. The light signal, and hence the amount of isoluminol-antibody conjugate, was measured by a photomultiplier in relative light units (RLU) and was indicative of the antibodies to SARS-CoV-2 present in the calibrators, samples, or controls. The Analyzer automatically calculated the SARS-CoV-2 IgG antibody levels expressed as arbitrary units per milliliter (AU/mL). Owing to the correlation of the results of this CLIA IgG to the values and units of the first WHO International Standard (IS) for anti-SARS-CoV-2 immunoglobulin binding activity [11], the AU/mL was converted to BAU/mL through multiplication by a factor of 2.6. The quantification range for the test was 1.85–800 AU/mL (4.81–2080 BAU/mL). According to the manufacturer’s recommendations, the samples with IgG titers <13.0 AU/mL, i.e., <33.8 BAU/mL, were considered as seronegative, while those with titers ≥13.0 AU/mL, i.e., ≥33.8 BAU/mL, were considered as seropositive to SARS-CoV-2. The sensitivity of this CLIA test, in cases when it is used 15 days after symptom onset, is 98.7%. The specificity of this test was 99.5% [12].

### 2.3. Data Analysis

The seroprevalence was a dichotomous outcome measure (seropositive or seronegative). The proportions of the IgG positive results (seropositive) obtained by the ELISA and CLIA tests in the analysis samples were calculated. A test of proportion was then performed to compare the values of seropositivity with the observed variables given by the ELISA and CLIA tests. Univariable and corrected multivariable logistic regression analyses were performed using seropositivity as the outcome variable. As the reference groups, we used the lowest values of seroprevalence regarding certain variables. The stratum seroprevalence and 95% confidence intervals (CIs) of SARS-CoV-2 seropositivity were calculated using the SPSS software tool (version 22.0) and the MedCalc for Windows, version 12.3.0 (MedCalc Software, Mariakerke, Belgium). The statistical significance was set at *p* < 0.05.

The clinical presentations of the COVID-19 disease (asymptomatic, mild, severe, and critical) were determined as previously described in detail [13].

### 2.4. Ethical Considerations

The study protocol, participant information form, and written informed consent form were reviewed and approved by the Ethics Committee of the Institute of Public Health of Vojvodina, Novi Sad (28 March 2022, number: 01-200/58-1). Participation in the study was based on a voluntary basis. Every participant was given the option to refuse to participate or to terminate the interview and their participation at any time during the study. All of the participants provided written informed consent. None of the authors of this study were involved in the treatment of the patients included in the analysis, and all data were anonymized before the authors accessed it.

## 3. Results

**Figure 1:**
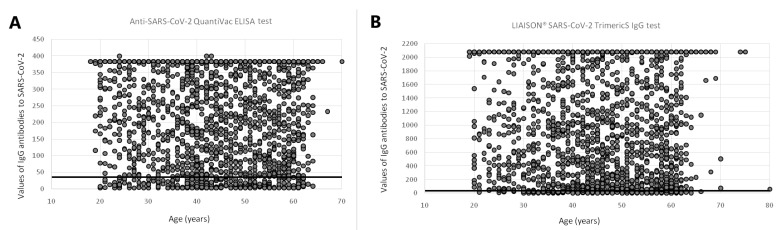
Scatter plot of the SARS-CoV-2 IgG concentrations using (**A**) Anti-SARS-CoV-2 QuantiVac ELISA test, and (**B)** LIAISON^®^ SARS-CoV-2 TrimericS IgG test, by age of healthcare workers in Vojvodina, Serbia. Legend: Horizontal line on panel (**A**) indicates the limit of reference level of protective antibodies (35.2 BAU/mL). Horizontal line on panel (**B**) indicates the limit of reference level of protective antibodies (33.8 BAU/mL).

In total, 6936 participants provided a blood sample along with the questionnaire. A total of 3835 (55.3%) participants were tested by the ELISA test, and 3101 (44.7%) by the CLIA test. The mean age of all of the participants was 45.62 years (median age 46 years; IQR 38–55 years). The mean age of the participants tested by the ELISA test was 44.94 years (median age 46 years; IQR 37–55 years), and there were 298 (7.77%), 1070 (27.90%), and 2467 (64.33%) serum samples from participants with antibody levels ≤35.2 BAU/mL, in the range between 35.2 and 380 BAU/mL, and >380 BAU/mL (above the limit of detection of the measured antibody level), respectively (Figure 1A). 

The mean age of the participants tested by the CLIA test was 46.46 years (median age 47 years; IQR 39–56 years), and there were 190 (6.13%), 1237 (39.89%), and 1674 (53.98%) serum samples from participants with antibody levels ≤33.8 BAU/mL, in the range between 33.8 and 2000 BAU/mL, and >2000 BAU/mL, respectively (Figure 1B).

Out of the 6936 tested participants, most of them were female (83.52%), aged 40–49 years (30.03%), nurses (46.90%), employed at the primary healthcare level (40.70%), without pre-existing medical conditions (77.80%), without previous contact with COVID-19 patients at the workplace (59.46%), with previous laboratory confirmation of COVID-19 (56.30%), and those who were vaccinated with at least one dose of the vaccine against COVID-19 (79.08%).

Overall, 6448 of the 6936 subjects (92.96%, 95% CI 92.33–93.55) tested positive for the presence of SARS-CoV-2-specific antibodies. More precisely, 92.23% (95% CI 91.34–93.06), and 93.87% (95% CI 92.97–94.69) of the serum samples were positive by the ELISA and CLIA tests for SARS-CoV-2-specific IgG antibodies, respectively, and this observed difference of seroprevalence was statistically significant (*p* = 0.0079). Significantly higher levels of seropositivity obtained by the CLIA test compared with the ELISA test were also observed among females (*p* = 0.0082), participants aged 18–29 (*p* = 0.0007) and 40–49 (*p* = 0.0061) years, among HCWs employed at the primary health care level (*p* = 0.0101), in those who did not have contact with COVID-19 patients at the workplace (*p* = 0.0282), among participants who had laboratory-confirmed COVID-19 cases (*p* = 0.0063), and in those who had not been vaccinated against COVID-19 (*p* = 0.0043) (Table 1).

Univariate and multivariate analyses were undertaken using the seropositivity to SARS-CoV-2 as the dependent variable and incorporating the participants’ age, gender, occupation, and the healthcare level of their workplace as the independent variables. There were numerous potential factors that might have influenced the seropositivity to SARS-CoV-2 antibodies in the studied population. Therefore, among the participants tested by the ELISA test, the seropositivity was significantly higher in the subjects with hypertension (98.77%) compared to the participants with malignancy (88%), and in those who had contact with COVID-19 patients at the workplace compared with those who did not, while among those tested by the CLIA test, the seropositivity to SARS-CoV-2 was significantly higher in participants aged 18–29 (98.44%) and 40–49 years (94.31%) compared with those aged 30–39 years (91.26%), as well as, among physicians (96.63%) in comparison with the laboratory technicians (91.18%). After adjusting, the chance of being seropositive was above two times higher among participants who had laboratory confirmation of COVID-19 and above 11 times higher among those who had been vaccinated with at least one dose of the SARS-CoV-2 vaccine compared to their counterparts, as measured by both serological tests (Table 2).

**Figure 2:**
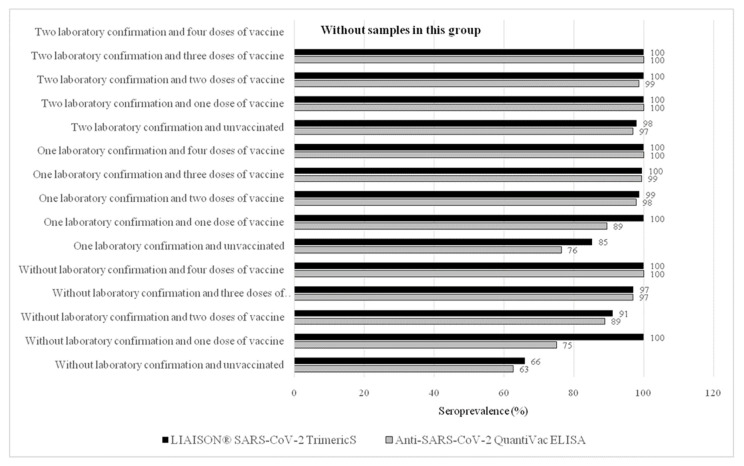
Seroprevalence of the SARS-CoV-2 antibodies regarding COVID-19 and vaccination status by two serological tests.

When we combined the data about previous SARS-CoV-2 infections and vaccination against COVID-19, the seropositivity to the SARS-CoV-2 virus significantly (*p* < 0.0001) increased with the number of SARS-CoV-2 infections and the number of doses of the SARS-CoV-2 vaccines received. Higher seropositivity was observed in HCWs with hybrid immunity, i.e., those who had previous infection(s) and were also vaccinated, emphasizing the importance of the vaccination of convalescents from a SARS-CoV-2 infection. In contrast, there were no significant differences in seropositivity among the participants with two laboratory confirmations of COVID-19, regardless of their vaccination status, and the number of received vaccines against COVID-19, because almost all of the subjects in these groups were seropositive (*p* > 0.005) (Figure 2, Table S1).

**Figure 3:**
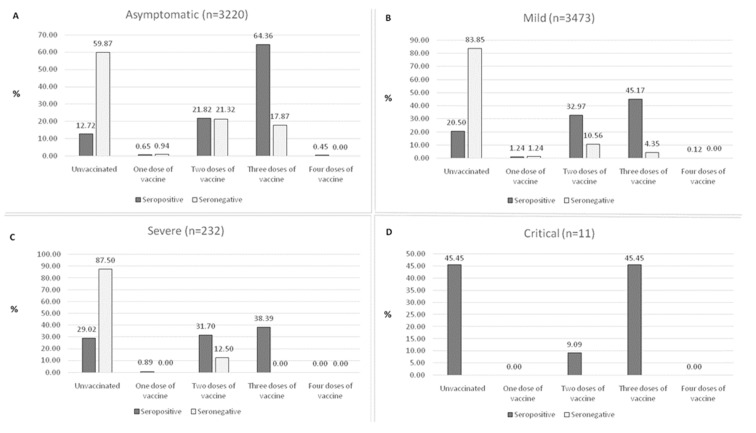
Seroprevalence of the SARS-CoV-2 antibodies in HCWs regarding the clinical form of their last episode of COVID-19: (**A**) asymptomatic, (**B**) mild, (**C**) severe, and (**D**) critical.

When the levels of antibodies were compared with regard to the clinical form of their last (most recent) COVID-19 episode and their vaccination status, the highest seroprevalence of SARS-CoV-2 antibodies was observed in participants with asymptomatic (64.36%), mild (45.17%), severe (38.39%), and critical (45.45%) forms who received three doses of the COVID-19 vaccines, as well as in unvaccinated participants who had and recovered from the critical form of COVID-19 (45.45%). On the other hand, with the exception of the critical form of COVID-19 (there were no seronegative samples to SARS-CoV-2 in this group), the highest seronegativity to SARS-CoV-2 was noticed among the unvaccinated participants regardless of the clinical form of COVID-19 they had (asymptomatic, 59.87%; mild, 83.85%; and severe, 87.50%) (Figure 3 A-D).

## 4. Discussion

In this cross-sectional study of asymptomatic people employed in healthcare centers of all three levels (primary, secondary, and tertiary) that covered 1/4 of the entire population of HCWs in Vojvodina, the overall SARS-CoV-2 seroprevalence was high (92.96%). The highest seroprevalence rates were found in those HCWs who had previous infection(s) and were also vaccinated, suggesting that such hybrid immunity could confer better protection against subsequent infections.

Previously published data from a research conducted among HCWs from three University Hospitals in Belgrade, Serbia, during the second wave of the COVID-19 pandemic (from June to early October, 2020, and before the start of the COVID-19 immunization in Serbia), showed that the overall seroprevalence of SARS-CoV-2 was only 18.3%. Specifically, the seropositivity among HCWs who worked in the COVID-19 hospital was higher (28.6%) compared with those who worked in the emergency center (12.6%) or those from non-COVID-19 hospitals (18.3%) [14]. In concordance with the aforementioned differences of seroprevalence, a longitudinal study among HCWs in Chile during the three-month period from April to July 2020, before and after the first wave of the COVID-19 pandemic, reported that the initial overall IgG seroprevalence of SARS-CoV-2 was 9.6%. However, the final cumulative value (after the first pandemic wave) in this Chilean study was 24% [15]. Thus, the seropositivity to SARS-CoV-2 may noticeably fluctuate depending on different parameters such as geographical location and the time when the research was conducted (stage of the pandemic), timeliness and the enforcement of infection control measures, the sampling strategies and type of serological test used, and may progressively increase over time. Therefore, it is not surprising that in our study, which was conducted later during the pandemic, after the sixth epidemic wave of COVID-19 and more than one year since the beginning of the vaccination campaigns in Serbia and elsewhere, the observed prevalence was much higher compared to the prevalence rates obtained in various populations of HCWs in several other SARS-CoV-2 seroprevalence studies that were performed before the period of our research [2,16,17,18,19,20,21,22,23,24,25]. In addition, numerous previous studies reported that the seroprevalence to SARS-CoV-2 among HCWs was higher in comparison with the general population [4,17,26,27]. Thus, the main reasons for these discrepancies of seroprevalence lie in a significant and repeated occupational exposures to the SARS-CoV-2 virus among HCWs during the COVID-19 pandemic [4] as well as higher vaccination coverage rates (VCR) in HCWs than in the general population. Indeed, VCR were estimated to be around 70% in HCWs compared to approximately 45% in the general population in Serbia [28].

As already mentioned, seroprevalence research can identify the missing COVID-19 cases with mild or no symptoms at all, that were left undiagnosed [15,16,18,19]. Our previous study of four consecutive rounds of surveys in asymptomatic individuals from the general population showed marked differences in the numbers of infections estimated by the survey and the officially reported cases in Vojvodina and gave insight into the proportion of the susceptible population in a community (over 80% of the total population) after two pandemic waves [2]. Although the seroprevalence rate of around 93% found in this study would imply that the number of seropositive HCWs could be around 25,796 out of the total 27,738 HCWs in Vojvodina, which is much more than the 19,272 officially reported cases of COVID-19 among HCWs (both primary infections and reinfections) until the end of the study, the same conclusion of the under-diagnosing and under-reporting of COVID-19 cannot be drawn since many of the HCWs might have become seropositive due to vaccination only, without being exposed to SARS-CoV-2. 

In the sample, the mean age of HCWs was 46 years, and the majority (78%) of them had no comorbidities. In addition, we found a variation in the seropositivity of SARS-CoV-2 among the different groups of subjects employed in the various healthcare centers in Vojvodina. Depending on the type of serological test used, the highest seroprevalence for participants tested by the ELISA test was in dentists (100%) and other medical staff (96.4%), while for those tested by the CLIA test the highest seropositivity of SARS-CoV-2 was observed in pharmacists (100%) and dentists (96.9%). The seroprevalence of SARS-CoV-2 in non-medical staff was 89.7% (ELISA test) and 92.1% (CLIA test). Although the observed differences might be due to the different performances of the two tests and/or the differences between the subjects in the two groups tested, it is also possible that they do reflect the specificities of the work of the different profiles of HCWs and a higher or lower risk of being exposed to the SARS-CoV-2. In line with this, the results of a previous published study showed that in comparison with the seroprevalence of other HCWs, the lower seroprevalence of SARS-CoV-2 was noticed among the participants working in intensive care medicine. This could be due to the fact that intensive care units were marked as units with high-risk environments where enhanced personal protective equipment was implemented [4]. Although we did not predict dividing the participants regarding the specificities of their workplaces, the seroprevalences in primary, secondary, and tertiary healthcare levels in our study were similar: 91.5%, 92.5%, and 92.6% (ELISA test) and 94.1%, 92.8%, and 94.3% (CLIA test), respectively. However, a multiple logistic regression model found a significantly higher risk of seropositivity to SARS-CoV-2 in participants who had contact with COVID-19 patients at the workplace (frontliners who were directly involved in diagnosing, treating, and caring for COVID-19 patients), which strongly supports the notion that the differential risk of SARS-CoV-2 depends on the type of occupational exposure at the workplace. Similar results were found by other authors [19,23].

It is well established that natural and vaccine-induced immunity to SARS-CoV-2 may have different mechanisms [29], and the increasing amount of data have been accumulated indicating higher levels of anti-SARS-CoV-2 antibodies (especially those against the S protein) in participants with combined natural/vaccine induced immunity, i.e., hybrid immunity. In this regard, the analysis of the factors associated with the seropositivity in HCWs in our study revealed that the participants who previously had laboratory-confirmed COVID-19 and/or those who have been vaccinated with at least one dose of the SARS-CoV-2 vaccine had a higher probability of being seropositive than their comparison groups. We further analyzed the probability for the seropositivity regarding the number of previous laboratory confirmations of COVID-19 along with the number of SARS-CoV-2 vaccines received. Shortly, the number of SARS-CoV-2 vaccines increased the seroprevalence of SARS-CoV-2 regardless of previous COVID-19 status (without laboratory confirmation or with one or two laboratory confirmations of COVID-19). Whether our findings mean better protection in those HCWs against new SARS-CoV-2 variants remains unclear, but a recently published systematic review on the efficacy and duration of natural and hybrid immunity shown that natural immunity has similar effect sizes regarding protection against reinfection across different SARS-CoV-2 variants, with the exception of the Omicron variant (data are just emerging before conclusions can be drawn) and that hybrid immunity appears to be the most protective against reinfections and, more importantly, against serious COVID-19 outcomes [30]. Nevertheless, the assessment of hybrid immunity in HCWs and its protective effects against the infection with Omicron subvariants was beyond the scope of the current study, and it is on future studies to delineate this.

In this study, we also found that some other factors were independently associated with the seropositivity of the participants, especially for those tested with the CLIA test. Although the reason for this remains unknown, similarly to results of other authors [31], we found that the participants aged 18–29 and 40–49 years had significantly higher odds of being seropositive than their older/younger counterparts. In general, all the observed differences in the seroprevalences obtained by the two serological tests could be interpreted as results of a different type and clinical performances of these tests, where the CLIA test is more sensitive in comparison with an ELISA test [10,12]. In other words, these differences were probably due to different performances of those assays and they do not represent true differences in the seroprevalence of the studied subpopulations.

In addition, our results support the previous findings that have correlated the severity of COVID-19 with the magnitude of the antibody response that follows the infection [13,32], i.e., seropositivity was significantly higher in individuals with prior symptomatic illness and those who have been vaccinated compared with those who remained asymptomatic or unvaccinated. It remains unclear which level of the antibodies against SARS-CoV-2 correlate with effective protection and further investigations should be done in order to determine the neutralizing capacity of the antibody responses associated with different severities of the disease/vaccination status, i.e., to measure the titres at which the neutralizing antibodies provide protection against infection and, if possible, also the duration of that protection [4].

Interestingly, the seropositivity of SARS-CoV-2 infections in the examined population was similar regardless of the comorbidities that some participants had when compared to those without any comorbidities. Although, there is the possibility that some of the participants were not fully aware of their comorbidity status, still, our results might be the base for more comprehensive research.

This study had some limitations. First, considering the fact that the participants self-presented to enroll, such an approach may have introduced a bias in the study cohort. Second, since we were not able to further dilute the sera with the available equipment support, we were not able to determine the average (median) values of seropositivity using the two different serological tests. Third, based on our data we could not distinguish whether SARS-CoV-2 infections were community or occupationally derived. We did not predict collecting personal data related to crowded workplaces, public transportation use, the appropriate use of masks during and outside of work, housing conditions as well as physical distancing history, and therefore we cannot be sure to what extent the workplace had a role in acquiring an infection by HCWs. However, it should be noted that the aim of this study was not to determine the efficacy of implemented non-pharmaceutical measures for the prevention of the spread of SARS-CoV-2, but we aimed to observe the immunity in HCWs considering the all-potential contact with the variants of SARS-CoV-2 before the advent of Omicron BA.4/BA.5. Fourth, although our serosurvey was conducted at the time when our country reported a decline in daily new cases of SARS-CoV-2, exclusion of symptomatic HCWs at the time point of the study might have slightly decreased the observed prevalence of anti-SARS-CoV-2 antibodies. Despite the above-mentioned potential limitations, we presume that they did not substantially compromise the main results of our study considering that we involved a large sample size of HCWs employed at various healthcare centers across Vojvodina. Finally, further longitudinal studies should be conducted to demonstrate the persistence of the current seropositivity, especially after the end of the actual epidemic wave caused by the Omicron sublineages of the SARS-CoV-2 virus.

## 5. Conclusions

In the present study we observed a high seropositivity levels of SARS-CoV-2 antibodies among HCWs in Vojvodina, a northern province of Serbia, during the Omicron predominance, before the advent of Omicron BA.4/BA.5 subvariants. The highest seroprevalence rates were found in those HCWs who had previous infection(s) and were also vaccinated, but it remains unknown whether this hybrid immunity could translate into protection against the new variants of concern. In order to better understand the level of immunity to actual variants of SARS-CoV-2 and to plan interventions efficiently, the vaccination coverage and seroprevalence of SARS-CoV-2 in both the risk groups and the general community must be assessed in future perspectives [33]. Although our results in HCWs cannot be directly extrapolated to other population groups in Vojvodina and Serbia, it is reasonable to assume that the seroprevalence rates in the general population in our country are relatively high; future studies are warranted to delineate this. In addition, the results of our recent study, which covered the period from the first confirmed COVID-19 case (6 March 2020) in Serbia to 31 January 2022, showed that SARS-CoV-2 reinfections in the general community were uncommon until the end of 2021, but became common with the emergence of Omicron and increased substantially thereafter [34]. Taking into account the results of our study, further research should evaluate the rates of reinfections and the associated severity of COVID-19 caused by the Omicron sublineages and/or new variants of the SARS-CoV-2 virus as well as the changes of the seroprevalence over time among HCWs who previously had natural and/or vaccine-induced immunity. 

## Figures and Tables

**Table 1:** Characteristics of total study population and stratified for their serostatus.

	All Participants			Anti-SARS-CoV-2 QuantiVac ELISA								LIAISON^®^ SARS-CoV-2 TrimericS								*p*-Value ^1^
				Total		Seroposi-tive		Seronegative		Seroprevalence % (95% CI)		Total		Seroposi-tive		Seronega-tive		Seroprevalence % (95% CI)		
	n = 6936			n = 3835		n = 3537		n = 298				n = 3101		n = 2911		n = 190				
	n	%	(95% CI)	n	%	n	%	n	%	92.23	91.34–93.06	n	%	n	%	n	%	93.87	92.97–94.69	0.0079
**Gender**																				
Male	1143	16.48	15.56–17.32	606	15.80	561	15.86	45	15.10	92.57	90.18–94.53	537	17.32	502	17.24	35	18.42	93.48	91.05–95.42	0.5475
Female	5793	83.52	82.63–84.39	3229	84.20	2976	84.14	253	84.90	92.16	91.18–93.06	2564	82.68	2409	82.76	155	81.58	93.95	92.96–94.84	**0.0082**
**Age (year)**																				
18–29	707	10.19	9.49–10.93	450	11.73	416	11.76	34	11.41	92.44	89.60–94.71	257	8.29	253	8.69	4	2.11	98.44	96.06–99.57	**0.0007**
30–39	1347	19.42	18.49–20.37	798	20.81	734	20.75	64	21.48	91.98	89.87–93.77	549	17.70	501	17.21	48	25.26	91.26	88.58–93.49	0.6383
40–49	2083	30.03	28.95–31.12	1099	28.66	1002	28.33	97	32.55	91.17	89.33–92.78	984	31.73	928	31.88	56	29.47	94.31	92.67–95.67	**0.0061**
50–59	2043	29.46	28.39–30.55	1098	28.63	1025	28.98	73	24.50	93.35	91.71–94.75	945	30.47	887	30.47	58	30.53	93.86	92.13–95.30	0.6391
≥60	756	10.90	10.18–11.66	390	10.17	360	10.18	30	10.07	92.31	89.20–94.75	366	11.80	342	11.75	24	12.63	93.44	90.40–95.75	0.5468
**Occupation**																				
Physician	1241	17.89	16.99–18.81	617	16.09	588	16.62	29	9.73	95.30	93.32–96.83	624	20.12	603	20.71	21	11.05	96.63	94.90–97.90	0.2338
Nurse	3253	46.90	45.72–48.08	1917	49.99	1766	49.93	151	50.67	92.12	90.82–93.29	1336	43.08	1253	43.04	83	43.68	93.79	92.36–95.02	0.0698
Pharmacist	70	1.01	0.79–1.27	42	1.10	37	1.05	5	1.68	88.10	74.37–96.02	28	0.90	28	0.96	0	0.00	100	87.66–100	0.0600
Dentist	93	1.34	1.08–1.64	29	0.76	29	0.82	0	0.00	100	88.06–100	64	2.06	62	2.13	2	1.05	96.88	89.17–99.62	0.3389
Laboratory technician	411	5.93	5.39–6.51	207	5.40	192	5.43	15	5.03	92.75	88.33–95.88	204	6.58	186	6.39	18	9.47	91.18	86.42–94.69	0.5586
Other medical staff	221	3.19	2.79–3.63	111	2.89	107	3.03	4	1.34	96.40	91.04-99.01	110	3.55	102	3.50	8	4.21	92.73	86.18-96.81	0.2296
Support non-medical staff	1647	23.75	22.75-24.77	912	23.78	818	23.13	94	31.54	89.69	87.53-91.59	735	23.70	677	23.26	58	30.53	92.11	89.92-93.95	0.0918
**Healthcare level**																				
Primary	2823	40.70	39.54-41.87	1158	30.20	1060	29.97	98	32.89	91.54	89.79-93.08	1665	53.69	1566	53.80	99	52.11	94.05	92.80-95.14	**0.0101**
Secondary	2026	29.21	28.14-30.30	1443	37.63	1334	37.72	109	36.58	92.45	90.96-93.76	583	18.80	541	18.58	42	22.11	92.80	90.39-94.76	0.7860
Tertiary	2087	30.09	29.01-31.18	1234	32.18	1143	32.32	91	30.54	92.63	91.03-94.02	853	27.51	804	27.62	49	25.79	94.26	92.48-95.72	0.1434
**Comorbidities**																				
Hypertension	186	2.68	2.31-3.09	81	2.11	80	2.26	1	0.34	98.77	93.32-99.97	105	3.39	101	3.47	4	2.11	96.19	90.53-98.95	0.2819
Chronic pulmonary disease	210	3.03	2.64-3.46	119	3.10	106	3.00	13	4.36	89.08	82.05-94.06	91	2.93	87	2.99	4	2.11	95.60	89.12-98.79	0.0868
Cardiovascular disease	441	6.36	5.80-6.96	253	6.60	230	6.50	23	7.72	90.91	86.67-94.15	188	6.06	179	6.15	9	4.74	95.21	91.10–97.79	0.0855
Diabetes	192	2.77	2.40–3.18	106	2.76	101	2.86	5	1.68	95.28	89.33–98.45	86	2.77	78	2.68	8	4.21	90.70	82.49–95.90	0.2103
Obesity	32	0.46	0.31–0.65	13	0.34	12	0.34	1	0.34	92.31	63.97–99.81	19	0.61	19	0.65	0	0.00	100	82.35–100	0.2268
Malignancy	90	1.30	1.05–1.60	50	1.30	44	1.24	6	2.01	88.00	75.69–95.47	40	1.29	38	1.31	2	1.05	95.00	83.08–99.39	0.2489
Other chronic disease	389	5.61	5.08–6.18	214	5.58	196	5.54	18	6.04	91.59	87.03–94.94	175	5.64	162	5.57	13	6.84	92.57	87.63–95.98	0.7229
Without comorbidity	5396	77.80	76.80–78.77	2999	78.20	2768	78.26	231	77.52	92.30	91.29–93.23	2397	77.30	2247	77.19	150	78.95	93.74	92.69–94.68	0.3382
**Contact with COVID-19 patients at workplace**																				
Yes	2812	40.54	39.38–41.71	1550	40.42	1448	40.94	102	34.23	93.42	92.07–94.60	1262	40.70	1196	41.09	66	34.74	94.77	93.39–95.93	0.1331
No	4124	59.46	58.29–60.62	2285	59.58	2089	59.06	196	65.77	91.42	90.20–92.54	1839	59.30	1715	58.91	124	65.26	93.26	92.02–94.36	**0.0282**
**Previously having laboratory-confirmed COVID-19**																				
Yes	3905	56.30	55.12–57.47	2170	56.58	2054	58.07	116	38.93	94.65	93.62–95.56	1735	55.95	1674	57.51	61	32.11	96.48	95.50–97.30	**0.0063**
No	3031	43.70	42.53–44.88	1665	43.42	1483	41.93	182	61.07	89.07	87.47–90.53	1366	44.05	1237	42.49	129	67.89	90.56	88.88–92.06	0.1786
**Vaccinated against COVID-19 with at least one dose of vaccine**																				
Yes	5485	79.08	78.10–80.03	3063	79.87	2965	83.83	98	32.89	96.80	96.11–97.39	2422	78.10	2365	81.24	57	30.00	97.65	96.97–98.22	0.0592
No	1451	20.92	19.97–21.90	772	20.13	572	16.17	200	67.11	74.09	70.85–77.15	679	21.90	546	18.76	133	70.00	80.41	77.22–83.33	**0.0043**

Values that differ significantly (*p* < 0.05) are marked in bold. ^1^ Test proportion differences between seroprevalence obtained by two different serological tests.

**Table 2:** Exposure factors associated with SARS-CoV-2 seropositivity stratified by univariable and multivariable logistic regression models.

	Anti-SARS-CoV-2 QuantiVac ELISA				LIAISON^®^ SARS-CoV-2 TrimericS			
	Crude OR (95% CI)	*p*-Value	Adjusted OR ^a,b^ (95% CI)	*p*-Value ^b^	Crude OR (95% CI)	*p*-Value	Adjusted OR ^a,b^ (95% CI)	*p*-Value ^b^
Gender								
Male	1.06 (0.76–1.47)	0.7297			Referent			
Female	Referent				1.08 (0.74–1.58)	0.6781		
Age (year)								
18–29	1.18 (0.79–1.78)	0.4150			6.06 (2.16–16.99)	**0.0006**	6.22 (2.21–17.47)	**0.0005**
30–39	1.11 (0.80–1.54)	0.5341			Referent			
40–49	Referent				1.59 (1.06–2.34)	**0.0237**	1.57 (1.05–2.36)	**0.0274**
50–59	1.36 (0.99–1.86)	0.0569			1.47 (0.98–2.18)	0.0598		
≥60	1.17 (0.76–1.78)	0.4913			1.367 (0.82–2.27)	0.2305		
Occupation								
Physician	2.74 (1.00–7.49)	0.0495			2.78 (1.45–5.33)	**0.0021**	2.72 (1.39–5.34)	**0.0036**
Nurse	1.58 (0.61–4.08)	0.3443			1.46 (0.86–2.49)	0.1628		
Pharmacist	Referent				5.65 (0.33–96.45)	0.2313		
Dentist	8.65 (0.46–162.87)	0.1496			3.00 (0.68–13.30)	0.1481		
Laboratory technician	1.73 (0.59–5.05)	0.3162			Referent			
Other medical staff	3.61 (0.92–14.18)	0.0654			1.23 (0.52–2.94)	0.6348		
Support non-medical staff	1.18 (0.45–3.07)	0.7402			1.13 (0.65–1.96)	0.6659		
Healthcare level								
Primary	Referent				1.23 (0.84–1.78)	0.2816		
Secondary	1.13 (0.85–1.50)	0.3948			Referent			
Tertiary	1.16 (0.86–1.56)	0.3244			1.27 (0.83–1.95)	0.2658		
Comorbidities								
Hypertension	10.91 (1.27–93.54)	**0.0293**	3.98 (0.41–38.53)	0.2336	2.59 (0.75–8.91)	0.1313		
Chronic pulmonary disease	1.11 (0.40–3.11)	0.8399			2.23 (0.65–7.70)	0.2042		
Cardiovascular disease	1.36 (0.52–3.54)	0.5243			2.04 (0.76–5.48)	0.1576		
Diabetes	2.75 (0.80–9.50)	0.1088			Referent			
Obesity	1.64 (0.18–14.93)	0.6624			4.22 (0.23–76.37)	0.3294		
Malignancy	Referent				1.95 (0.39–9.63)	0.4130		
Other chronic disease	1.48 (0.56–3.96)	0.4292			1.28 (0.51–3.21)	0.6016		
Without comorbidity	1.63 (0.69–3.87)	0.2650			1.54 (0.73–3.24)	0.2593		
Contact with COVID-19 patients at workplace								
Yes	1.33 (1.04–1.71)	**0.0238**	1.46 (1.13–1.90)	**0.0041**	1.31 (0.96–1.78)	0.0852		
No	Referent				Referent			
Previously having laboratory-confirmed COVID-19								
Yes	2.17 (1.71–2.77)	**<0.0001**	2.18 (1.71–2.78)	**<0.0001**	2.86 (2.09–3.91)	**<0.0001**	2.90 (2.12–3.97)	**<0.0001**
No	Referent				Referent			
Vaccinated against COVID-19 with at least one dose of vaccine								
Yes	10.58 (8.18–13.69)	**<0.0001**	11.34 (8.70–14.78)	**<0.0001**	10.11 (7.31–13.97)	**<0.0001**	11.05 (7.92–15.40)	**<0.0001**
No	Referent				Referent			

The group with the lowest seropositivity by characteristics was used as the reference. ^a^ Adjusted for the following variables: age, gender, occupation, and healthcare level. ^b^ Only identified risk factors for seronegativity with a p-value <0.05 in the univariable analyses were included in the multivariable logistic regression analyses. Values that differ significantly (*p* < 0.05) are marked in bold.

## Data Availability

The data that support the findings of this study are available from corresponding authors upon reasonable request.

## Supplementary Materials

The following supporting information can be downloaded at: https://www.mdpi.com/article/10.3390/vaccines10122168/s1, Table S1: Seropositivity of participants according to the number of previous SARS-CoV-2 infections and/or number of COVID-19 vaccines received, as measured by two serological tests.

## References

[1] Wilson S.E., Deeks S.L., Hatchette T.F., Crowcroft N.S. (2012). The Role of Seroepidemiology in the Comprehensive Surveillance of Vaccine-Preventable Diseases. CMAJ.

[2] Ristić M., Milosavljević B., Vapa S., Marković M., Petrović V. (2021). Seroprevalence of Antibodies against SARS-CoV-2 Virus in Northern Serbia (Vojvodina): A Four Consecutive Sentinel Population-Based Survey Study. PLoS ONE.

[3] Lai C.-C., Wang J.-H., Hsueh P.-R. (2020). Population-Based Seroprevalence Surveys of Anti-SARS-CoV-2 Antibody: An up-to-Date Review. Int. J. Infect. Dis..

[4] Shields A., Faustini S.E., Perez-Toledo M., Jossi S., Aldera E., Allen J.D., Al-Taei S., Backhouse C., Bosworth A., Dunbar L.A. (2020). SARS-CoV-2 Seroprevalence and Asymptomatic Viral Carriage in Healthcare Workers: A Cross-Sectional Study. Thorax.

[5] Chen W.-Q., Lu C.-Y., Wong T.-W., Ling W.-H., Lin Z.-N., Hao Y.-T., Liu Q., Fang J.-Q., He Y., Luo F.-T. (2005). Anti-SARS-CoV Immunoglobulin G in Healthcare Workers, Guangzhou, China. Emerg. Infect. Dis..

[6] Ministry of Health of the Republic of Serbia Coronavirus COVID-19. https://covid19.rs/eng-coronavirus-analytics/.

[7] Pustahija T., Ristić M., Medić S., Vuković V., Štrbac M., Rajčević S., Patić A., Petrović V. (2021). Epidemiological Characteristics of COVID-19 Travel-Associated Cases in Vojvodina, Serbia, during 2020. PLoS ONE.

[8] Petrović V., Vuković V., Marković M., Ristić M. (2022). Early Effectiveness of Four SARS-CoV-2 Vaccines in Preventing COVID-19 among Adults Aged ≥ 60 Years in Vojvodina, Serbia. Vaccines.

[9] The Government of the Republic of Serbia COVID-19. https://www.srbija.gov.rs/sekcija/en/151926/covid-19.php.

[10] Euroimmun Medizinische Labordiagnostika AG Anti-SARS-CoV-2 QuantiVac ELISA IgG Lübeck, Germany: EuroimmunMedizinischeLabordiagnostika AG. https://www.coronavirus-diagnostics.com/documents/Indications/Infections/Coronavirus/EI_2606_D_UK_E.pdf.

[11] WHO First WHO International Standard for Anti-SARS-CoV-2 Immunoglobulin (Human). NIBSC Code: 20/136. Instructions for Use. Version 2.0..

[12] Diasorin S.p.A. LIAISON ® SARS-CoV-2 TrimericS IgG. Saluggia, Italy: Diasorin S.p.A. https://www.diasorin.com/sites/default/files/allegati_prodotti/liaisonr_sars-cov-2_trimerics_igg_assay_m0870004408_a_lr_0.pdf.

[13] Petrović V., Vuković V., Patić A., Marković M., Ristić M. (2022). Immunogenicity of BNT162b2, BBIBP-CorV and Gam-COVID-Vac Vaccines and Immunity after Natural SARS-CoV-2 Infection-A Comparative Study from Novi Sad, Serbia. PLoS ONE.

[14] Markovic-Denic L., Zdravkovic M., Ercegovac M., Djukic V., Nikolic V., Cujic D., Micic D., Pekmezovic T., Marusic V., Popadic V. (2022). Seroprevalence in Health Care Workers during the Later Phase of the Second Wave: Results of Three Hospitals in Serbia, Prior to Vaccine Administration. J. Infect. Public Health.

[15] Iruretagoyena M., Vial M.R., Spencer-Sandino M., Gaete P., Peters A., Delgado I., Perez I., Calderon C., Porte L., Legarraga P. (2021). Longitudinal Assessment of SARS-CoV-2 IgG Seroconversionamong Front-Line Healthcare Workers during the First Wave of the COVID-19 Pandemic at a Tertiary-Care Hospital in Chile. BMC Infect. Dis..

[16] Pagen D.M.E., Brinkhues S., Dukers-Muijrers N.H.T.M., den Heijer C.D.J., Bouwmeester-Vincken N., Hanssen D.A.T., van Loo I.H.M., Savelkoul P.H.M., Hoebe C.J.P.A. (2022). Exposure Factors Associated with SARS-CoV-2 Seroprevalence during the First Eight Months of the COVID-19 Pandemic in the Netherlands: A Cross-Sectional Study. PLoS ONE.

[17] Aryal S., Pandit S., Pokhrel S., Chhusyabaga M., Bista P., Bhatt M.P., Subedi D.D., Rijal B.P. (2022). Anti-SARS-CoV-2 Antibody Screening in Healthcare Workers and Its Correlation with Clinical Presentation in Tertiary Care Hospital, Kathmandu, Nepal, from November 2020 to January 2021. Interdiscip. Perspect. Infect. Dis..

[18] El-Sokkary R.H., Daef E., El-Korashi L.A., Khedr E.M., Gad D., Mohamed-Hussein A., Zayed N.E., Mostafa E.F., Bahgat S.M., Hassany S.M. (2021). Sero-Prevalence of Anti-SARS-CoV-2 Antibodies among Healthcare Workers: A Multicenter Study from Egypt. J. Infect. Public Health.

[19] Gelanew T., Seyoum B., Mulu A., Mihret A., Abebe M., Wassie L., Gelaw B., Sorsa A., Merid Y., Muchie Y. (2022). High Seroprevalence of Anti-SARS-CoV-2 Antibodies among Ethiopian Healthcare Workers. BMC Infect. Dis..

[20] Al-Naamani K., Al-Jahdhami I., Al-Tamtami W., Al-Amri K., Al-Khabori M., Al Sinani S., Said E.A., Omer H., Al-Bahluli H., Al-Ryiami S. (2021). Prevalence and Persistence of SARS-CoV2 Antibodies among Healthcare Workers in Oman. J. Infect. Public Health.

[21] Haq I., Qurieshi M.A., Khan M.S., Majid S., Bhat A.A., Kousar R., Chowdri I.N., Qazi T.B., Lone A.A., Sabah I. (2021). The Burden of SARS-CoV-2 among Healthcare Workers across 16 Hospitals of Kashmir, India-A Seroepidemiological Study. PLoS ONE.

[22] Wiggen T.D., Bohn B., Ulrich A.K., Stovitz S.D., Strickland A.J., Naumchik B.M., Walsh S., Smith S., Baumgartner B., Kline S. (2022). SARS-CoV-2 Seroprevalence among Healthcare Workers. PLoS ONE.

[23] Kasztelewicz B., Janiszewska K., Burzyńska J., Szydłowska E., Migdał M., Dzierżanowska-Fangrat K. (2021). Prevalence of IgG Antibodies against SARS-CoV-2 among Healthcare Workers in a Tertiary Pediatric Hospital in Poland. PLoS ONE.

[24] Alkurt G., Murt A., Aydin Z., Tatli O., Agaoglu N.B., Irvem A., Aydin M., Karaali R., Gunes M., Yesilyurt B. (2021). Seroprevalence of Coronavirus Disease 2019 (COVID-19) among Health Care Workers from Three Pandemic Hospitals of Turkey. PLoS ONE.

[25] Dávila-Conn V., Soto-Nava M., Caro-Vega Y.N., Paz-Juárez H.E., García-Esparza P., Tapia-Trejo D., Pérez-García M., Belaunzarán-Zamudio P.F., Reyes-Terán G., Sierra-Madero J.G. (2022). Seroepidemiology of SARS-CoV-2 in Healthcare Personnel Working at the Largest Tertiary COVID-19 Referral Hospitals in Mexico City. PLoS ONE.

[26] Houlihan C.F., Vora N., Byrne T., Lewer D., Heaney J., Moore D.A., Matthews R., Adam S., Enfield L., Severn A. (2020). SARS-CoV-2 Virus and Antibodies in Front-Line Health Care Workers in an Acute Hospital in London: Preliminary Results from a Longitudinal Study. medRxiv.

[27] Eyre D.W., Lumley S.F., O’Donnell D., Campbell M., Sims E., Lawson E., Warren F., James T., Cox S., Howarth A. (2020). Differential Occupational Risks to Healthcare Workers from SARS-CoV-2 Observed during a Prospective Observational Study. eLife.

[28] Institute of Public Health of Serbia “Dr Milan Jovanović Batut” Imunizacija [In Serbian]. https://www.batut.org.rs/index.php?category_id=186.

[29] Andreano E., Paciello I., Piccini G., Manganaro N., Pileri P., Hyseni I., Leonardi M., Pantano E., Abbiento V., Benincasa L. (2021). Hybrid Immunity Improves B Cells and Antibodies against SARS-CoV-2 Variants. Nature.

[30] Pilz S., Theiler-Schwetz V., Trummer C., Krause R., Ioannidis J.P.A. (2022). SARS-CoV-2 Reinfections: Overview of Efficacy and Duration of Natural and Hybrid Immunity. Environ. Res..

[31] Visci G., Zunarelli C., Mansour I., Porru S., De Palma G., Duval X., Monaco M.G.L., Spiteri G., Carta A., Lippi G. (2022). Serological Response after SARS-CoV2 Vaccination in Healthcare Workers: A Multicenter Study. Med. Lav..

[32] Zhao J., Yuan Q., Wang H., Liu W., Liao X., Su Y., Wang X., Yuan J., Li T., Li J. (2020). Antibody Responses to SARS-CoV-2 in Patients with Novel Coronavirus Disease 2019. Clin. Infect. Dis..

[33] Ara J., Islam M.S., Quader M.T.U., Das A., Hasib F.M.Y., Islam M.S., Rahman T., Das S., Chowdhury M.A.H., Das G.B. (2022). Sero-Prevalence of Anti-SARS-CoV-2 Antibodies in Chattogram Metropolitan Area, Bangladesh. medRxiv.

[34] Medić S., Anastassopoulou C., Lozanov-Crvenković Z., Vuković V., Dragnić N., Petrović V., Ristić M., Pustahija T., Gojković Z., Tsakris A. (2022). Risk and Severity of SARS-CoV-2 Reinfections during 2020-2022 in Vojvodina, Serbia: A Population-Level Observational Study. Lancet Reg. Health Eur..

