# Supplementary material for: Seroepidemiology of SARS-CoV-2 Virus in Healthcare Workers before Circulation of the Omicron Sublineages BA.4/BA.5 in Vojvodina, Serbia
Source: Vaccines (Basel). 2022 Dec 16;10(12):2168. doi: 10.3390/vaccines10122168 (PMC9783630; doi:10.3390/vaccines10122168)
Supplement: Supplementary file 1 [file vaccines-10-02168-s001.zip › vaccines-2039194-supplementary.pdf]

Table S1. Seropositivity of participants according to number of previous SARS-CoV-2 infection and/or number of COVID-19 vaccines received as measured by two serological tests.

**Variable**	Anti-SARS-CoV-2 QuantiVac ELISA						LIAISON® SARS-CoV-2 TrimericS					
	No of samples	Seropositive		Seronegative		p value	No of samples	Seropositive		Seronegative		p-value
		n	%	n	%			n	%	n	%	
Without laboratory confirmation and unvaccinated	284	178	62.68	106	37.32	< 0.0001	235	155	65.96	80	34.04	< 0.0001
Without laboratory confirmation and one dose of vaccine	12	9	75.00	3	25.00		9	9	100.00	0	0.00	
Without laboratory confirmation and two doses of vaccine	379	337	88.92	42	11.08		272	248	91.18	24	8.82	
Without laboratory confirmation and three doses of vaccine	984	953	96.85	31	3.15		844	819	97.04	25	2.96	
Without laboratory confirmation and four doses of vaccine	6	6	100.00	0	0.00		6	6	100.00	0	0.00	
One laboratory confirmation and unvaccinated	387	296	76.49	91	23.51	< 0.0001	345	294	85.22	51	14.78	< 0.0001
One laboratory confirmation and one dose of vaccine	19	17	89.47	2	10.53		17	17	100.00	0	0.00	
One laboratory confirmation and two doses of vaccine	587	574	97.79	13	2.21		406	401	98.77	5	1.23	
One laboratory confirmation and three doses of vaccine	799	794	99.37	5	0.63		665	662	99.55	3	0.45	
One laboratory confirmation and four doses of vaccine	2	2	100.00	0	0.00		3	3	100.00	0	0.00	
Two laboratory confirmation and unvaccinated	99	96	96.97	3	3.03	0.4139	97	95	97.94	2	2.06	0.3858
Two laboratory confirmation and	4	4	100.00	0	0.00		6	6	100.00	0	0.00	

one dose of vaccine												
Two laboratory confirmation and two doses of vaccine	140	138	98.57	2	1.43		98	98	100.00	0	0.00	
Two laboratory confirmation and three doses of vaccine	129	129	100.00	0	0.00		96	96	100.00	0	0.00	
Two laboratory confirmation and four doses of vaccine	0	0	0.00	0	0.00		0	0	0.00	0	0.00	
Three laboratory confirmation and unvaccinated	2	2	100.00	0	0.00		2	2	100.00	0	0.00	
Three laboratory confirmation and one dose of vaccine	0	0	0.00	0	0.00		0	0	0.00	0	0.00	
Three laboratory confirmation and two doses of vaccine	1	1	100.00	0	0.00	NA	0	0	0.00	0	0.00	NA
Three laboratory confirmation and three doses of vaccine	1	1	100.00	0	0.00		0	0	0.00	0	0.00	
Three laboratory confirmation and four doses of vaccine	0	0	0.00	0	0.00		0	0	0.00	0	0.00	
